# Computer-Aided Diagnosis of Laryngeal Cancer Based on Deep Learning with Laryngoscopic Images

**DOI:** 10.3390/diagnostics13243669

**Published:** 2023-12-14

**Authors:** Zhi-Hui Xu, Da-Ge Fan, Jian-Qiang Huang, Jia-Wei Wang, Yi Wang, Yuan-Zhe Li

**Affiliations:** 1 Department of Otolaryngology, The Second Affiliated Hospital, Fujian Medical University, 950 Donghai Street, Fengze District, Quanzhou 362000, China; xzh1212kk@126.com (Z.-H.X.);; 2 Department of Pathology, The Second Affiliated Hospital, Fujian Medical University, 950 Donghai Street, Fengze District, Quanzhou 362000, China; fjpathologyfdg@163.com; 3 Department of Emergency, The Second Affiliated Hospital, Fujian Medical University, 950 Donghai Street, Fengze District, Quanzhou 362000, China; wjw57900@163.com; 4 CT/MRI Department, The Second Affiliated Hospital, Fujian Medical University, 950 Donghai Street, Fengze District, Quanzhou 362000, China

**Keywords:** laryngeal cancer, deep learning, Densenet201, laryngoscopic images, computer-aided diagnosis, diagnostic accuracy

## Abstract

Laryngeal cancer poses a significant global health burden, with late-stage diagnoses contributing to reduced survival rates. This study explores the application of deep convolutional neural networks (DCNNs), specifically the Densenet201 architecture, in the computer-aided diagnosis of laryngeal cancer using laryngoscopic images. Our dataset comprised images from two medical centers, including benign and malignant cases, and was divided into training, internal validation, and external validation groups. We compared the performance of Densenet201 with other commonly used DCNN models and clinical assessments by experienced clinicians. Densenet201 exhibited outstanding performance, with an accuracy of 98.5% in the training cohort, 92.0% in the internal validation cohort, and 86.3% in the external validation cohort. The area under the curve (AUC) values consistently exceeded 92%, signifying robust discriminatory ability. Remarkably, Densenet201 achieved high sensitivity (98.9%) and specificity (98.2%) in the training cohort, ensuring accurate detection of both positive and negative cases. In contrast, other DCNN models displayed varying degrees of performance degradation in the external validation cohort, indicating the superiority of Densenet201. Moreover, Densenet201’s performance was comparable to that of an experienced clinician (Clinician A) and outperformed another clinician (Clinician B), particularly in the external validation cohort. Statistical analysis, including the DeLong test, confirmed the significance of these performance differences. Our study demonstrates that Densenet201 is a highly accurate and reliable tool for the computer-aided diagnosis of laryngeal cancer based on laryngoscopic images. The findings underscore the potential of deep learning as a complementary tool for clinicians and the importance of incorporating advanced technology in improving diagnostic accuracy and patient care in laryngeal cancer diagnosis. Future work will involve expanding the dataset and further optimizing the deep learning model.

## 1. Introduction

Head and neck tumors rank as the seventh most prevalent worldwide [1]. Laryngeal squamous cell cancer stands as the second most common subtype within head and neck squamous cell cancers, surpassed solely by oral squamous cell cancer [2,3]. According to the data of the World Cancer Report, 184,615 new cases of laryngeal cancer were diagnosed and 99,840 associated deaths were recorded worldwide in 2020 [4]. The burden of this disease is substantial. In China alone, 27,832 new cases of laryngeal cancer are diagnosed annually nationwide, resulting in 15,698 deaths [5]. The repercussions on people’s health and quality of life are profound [6].

Clinical outcomes in laryngeal squamous cell carcinoma (LSCC) are intricately linked to tumor stage. The specific survival rate for T1a stage cases is an encouraging 98.1%, while for T4 stage cases, it plummets to a mere 47.6% [7,8]. Given the inconspicuous early symptoms, approximately 60% of patients seek treatment only when their condition has advanced significantly, missing the window for optimal treatment. Thus, effective means for early detection, continuous monitoring, and accurate diagnosis are pivotal to improving treatment outcomes [9]. While puncture biopsy is an invasive examination, it remains the prevailing method. Nevertheless, given that histological confirmation is currently considered the cornerstone of cancer diagnosis, it is challenging to envision a future where biopsies are not necessary. Despite the fact that biopsy procedures and histologic analysis may not be entirely error-free, they continue to play a crucial role.

At present, narrow band imaging (NBI), which can enhance the visualization of microvascular patterns in the surface layer of the tissue, has played a crucial role in early LSCC detection, boasting high sensitivity (88.9–97.0%) and specificity (84.6–96.0%) [10,11,12,13]. However, the widespread adoption of NBI necessitates costly imaging equipment, specific training, and experienced endoscopists, constraining its applicability in many developing countries. Consequently, the use of traditional laryngoscopic images with white light endoscopy (WLE) carries practical significance, particularly in less developed regions with a shortage of experienced endoscopists. Moreover, the analysis shows that for artificial intelligence (AI), there are no statistically significant differences in the accuracy of differentiating benign and malignant lesions in the WLE and NBI [14].

Owing to the distinctive clinicopathologic features of laryngeal cancer lesions, identifying them through non-magnified endoscopy can be a formidable task for the human eye. In the 1960s, Hubel’s pioneering work [15] illuminated the neural network architecture of the cat cortex, leading to the development of convolutional neural networks (CNNs). CNNs are known for their significant advantage in processing large-scale images [16,17] and have emerged as a focal point of research in various scientific domains, including medicine. However, laryngeal squamous cell carcinoma (LSCC) remains underexplored in AI research [18,19,20,21,22,23,24,25,26]. Fortunately, deep convolutional neural networks (DCNNs) have recently exhibited remarkable diagnostic capabilities across various diseases, such as breast tumors and interstitial pulmonary disorders. In recent years, significant progress has been made in artificial intelligence research in the field of head and neck tumors [27,28,29]. Researchers have widely applied various artificial intelligence algorithms, promoting innovation in clinical diagnosis and treatment. Yin Wang et al. constructed predictive models using various artificial intelligence algorithms, which provide important assistance in the treatment efficacy, recurrence, and progression of head and neck tumors. Research on the application of AI artificial intelligence in clinical decision-making and prognostic analysis of head and neck tumors is constantly emerging [30]. The model based on CT imaging omics has achieved results in existing research in predicting the prognosis of nasopharyngeal carcinoma and the efficacy of radiotherapy and chemotherapy. The application of artificial intelligence in the field of ear, nose, throat, head and neck surgery has expanded to include tumor diagnosis, clinical decision support, and disease mechanism research, bringing new hope to public health. Regarding the recognition of anatomical sites in laryngoscopy images, Wang Meiling et al.’s research achieved automatic recognition and classification of anatomical sites in electronic laryngoscopy examination through an artificial intelligence quality control system based on convolutional neural networks. Overall, these studies provide strong support for early diagnosis, treatment decision-making, and prognosis analysis of head and neck tumors, highlighting the broad application prospects of artificial intelligence in the field of head and neck tumors [31].

Trained on extensive sets of images representing the diseases, a DCNN model learns through specific optimization algorithms. During testing and external validation phases, it autonomously predicts a given test or validation images [32,33]. Benefiting from DCNN’s robust texture features and training on large datasets, the model generalizes well to unseen testing images, often achieving comparable or superior classification accuracy compared to a specialist. Notably, CNNs have demonstrated particular aptitude in computer vision, particularly in image interpretation, spanning domains like skin and retinal diseases [34,35,36]. In this study, we posit that deep learning techniques can similarly enhance the clinical diagnosis of LSCC. To this end, we have amassed a substantial repository of laryngoscopic images to construct a DCNN model and evaluate its performance. This study innovates by introducing, for the first time, the application of Densenet201 in laryngeal cancer recognition. Additionally, it incorporates laryngoscopic data from various medical centers as the external validation group. The research employs a multi-model modeling approach, facilitating comprehensive comparisons. Notably, we also compare the top-performance of our deep learning model with the diagnostic capabilities of clinical experts. Through this multifaceted analysis, the study provides robust evidence supporting the efficacy of deep learning in enhancing the diagnostic accuracy of laryngeal cancer under laryngoscopy. In this article, we introduced the detailed process of the experiment in the Section 2, which includes the methods of material collection, model establishment, and model validation. In the Section 3, we provide a detailed introduction to our experimental results. Subsequently, in the Section 4, we conducted a detailed discussion based on clinical background and experimental results. Finally, we summarized the conclusion of the article in the Section 5.

## 2. Materials and Methods

**Figure 1:**
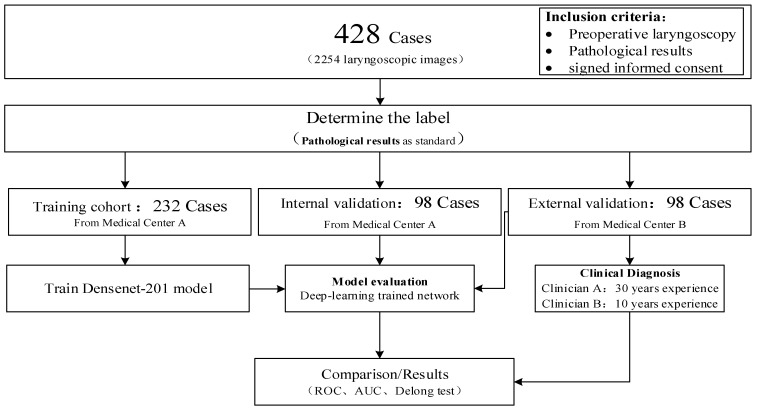
The experimental flowchart of this study.

The data for this study came from two medical centers and was divided into a training group, an internal validation group, and an external validation group. The specific experimental process is shown in Figure 1. Densenet201 was used to train a benign and malignant automatic discrimination model, and external validation groups were used to verify the model’s performance. At the same time, we compared it with other commonly used deep learning models. We invited a chief physician with over 30 years of experience in otolaryngology diagnosis and treatment—Clinician A—and an address physician with 10 years of experience in otolaryngology diagnosis and treatment—Clinician B—to evaluate the malignant risk of lesions in the external validation set of phonoscope images, with a risk value ranging from 0.00 to 1.00. We also draw ROC curves and calculate AUC values, and conduct a Delong test with the external validation group of our Densenet201 model.

### 2.1. Study Population and Imaging Acquisitions

**Figure 2:**
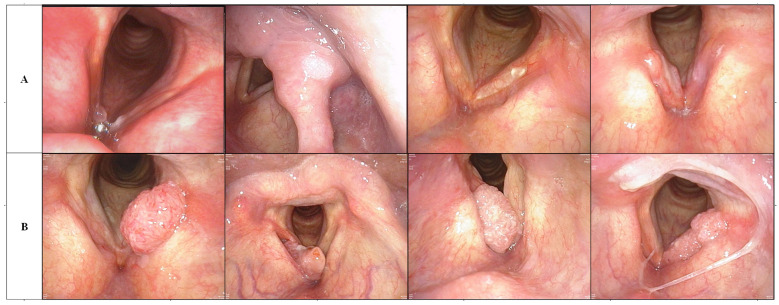
(**A**) Line is a benign laryngoscopy image, while line (**B**) is a malignant laryngoscopy image. The first image in line (**A**) is a polyp case, the second is a papilloma, the third is a tuberculosis, and the fourth is a granulomatous lesion.

Data were acquired from two medical centers. Medical center A is the Donghai Campus of the Second Affiliated Hospital of Fujian Medical University. Medical center B is the Licheng Campus of the Second Affiliated Hospital of Fujian Medical University. In this study conducted from January 2019 to June 2023, 428 patients with laryngeal lesions visited otolaryngology head and neck surgery departments at medical centers A and B. At medical center A, a simple randomization method was used to select 127 cases of benign laryngeal lesions (53 males and 74 females, aged 45 ± 12.3 years) and 105 cases of laryngeal squamous cell carcinoma (102 males and 3 females, aged 52 ± 8.6 years) for training and calibrating the AI system. The remaining cases at medical center A, comprising 53 males (20 males and 33 females, aged 46 ± 12.8 years) with benign laryngeal lesions and 45 cases of laryngeal squamous cell carcinoma (44 males and one female, aged 52 ± 9.6 years), underwent internal AI testing. The cases in medical center B were used as external testing, with 53 males (24 males and 29 females, aged 41 ± 11.2 years) with benign laryngeal lesions, 45 cases of laryngeal squamous cell carcinoma (44 males, one female, age 53 ± 9.1 years) underwent external testing of AI. Between January 2019 and June 2023, 195 cases with pathologically confirmed LSCC on surgical resection were retrieved. One hundred and fifty (150) cases from medical center A were used as a training and internal validation cohort and 45 cases from medical center B were used as an external validation cohort. Two hundred and thirty three (233) cases with pathologically confirmed benign lesions of larynx also were retrieved from two medical centers. One hundred and eighty (180) cases from medical center A were used as a training and internal validation cohort and 53 cases from medical center B were used as an external validation cohort. Our raw laryngoscopic images were captured using integration system endoscopes (CV-170, Olympus Medical Systems Corp., Tokyo, Japan) and standard endoscopes (OTV-S7, Olympus Medical Systems Corp., Tokyo, Japan), endoscopic systems (LMD-1420; Shanghai Suoguang Visual Products Corp., Shanghai, China and CLV-S40; Olympus Medical Systems Corp., Tokyo, Japan). An experienced endoscopist elected four to 11 high quality images from the raw images captured from different perspectives for each case for data augmentation and a total of 2254 laryngoscopic images were included in this study, including LSCC, benign laryngeal tumors such as polyps and non-specific inflammation and so on which were all biopsy-proven. Demographic and clinical characteristics were collected from the case management system, including age, gender, pathology and tumor size marked T (according to American Joint Committee on Cancer about LSCC) [24]. Patients from medical center A were divided randomly into training and internal validation cohorts with a ratio of 7:3. Patients from medical center B were utilized as the external validation cohort. A summary of the image sets and clinical characteristics were detailed provided in Table 1. In Figure 2, we present a set of examples of benign and malignant laryngoscopic images. Table 2 presents the histopathological results of the benign lesions encountered in our study. It is important to note that our dataset included a diverse range of benign lesions, including papilloma, tuberculosis, and granulomatous lesions, among others. The inclusion of these benign lesions allowed for a comprehensive assessment of the diagnostic performance of Densenet201 across various histopathological categories.

### 2.2. Structure of CNN Model

In this study, we have leveraged the power of the Densenet201 architecture, a state-of-the-art convolutional neural network (CNN), renowned for its outstanding performance in image recognition tasks. Densenet, short for Densely Connected Convolutional Networks, exhibits a unique architectural characteristic—dense connectivity. This feature sets it apart from traditional CNN architectures by establishing direct connections between layers within the network. Densenet201, an extension of the original Densenet architecture, is a deep convolutional neural network (CNN) that excels in image recognition tasks. It is particularly well-suited for extracting features from complex images [37,38]. Here is a breakdown of its key architectural components:

Dense Blocks: Densenet201 comprises multiple dense blocks, each containing a series of densely connected convolutional layers. In these blocks, each layer receives feature maps not just from the previous layer but also from all preceding layers within the same block. This dense connectivity promotes feature reuse, enabling the network to capture both low-level and high-level features effectively.

Transition Layers: Between dense blocks, transition layers are inserted. These layers include batch normalization, a pooling operation (typically average pooling), and a convolutional layer with a bottleneck structure (1 × 1 convolution). Transition layers reduce the spatial dimensions of feature maps while increasing the number of channels, striking a balance between computational efficiency and expressive power.

Global Average Pooling (GAP): At the end of the network, a global average pooling layer is used to aggregate the feature maps spatially, resulting in a single vector for each feature map. This reduces the spatial dimension to 1 × 1, enabling the network to produce a fixed-size feature vector regardless of input size.

Fully Connected Layer: Following GAP, a fully connected layer performs the final classification. The number of neurons in this layer corresponds to the number of classes in the classification task.

Feature Reuse: Densenet’s dense connectivity allows for maximum feature reuse, which facilitates the learning of more compact and discriminative representations from the data [35].

Mitigating Vanishing Gradient: The dense connections ensure the flow of gradients during training, mitigating the vanishing gradient problem often encountered in very deep networks.

Efficient Parameter Utilization: Densenet’s parameter-efficient design enables it to maintain high accuracy while using fewer parameters compared to traditional architectures [36].

State-of-the-Art Performance: Densen201 consistently achieves state-of-the-art performance in various image recognition challenges, outperforming many other architectures in terms of both accuracy and computational efficiency [39,40].

**Figure 3:**
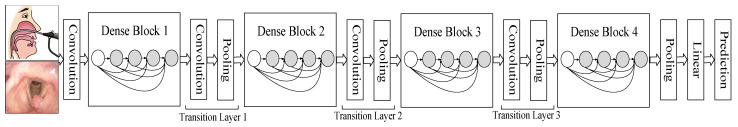
The network structure diagram of Densenet201.

The network structure diagram of Densenet201 and detailed parameters can be seen in Figure 3 and Table 3.

### 2.3. Training Process of DCNN Model

The hardware equipment utilized was the NVIDIA RTX 3090 24 G. The software environment incorporated Python 3.6, Pytorch 0.4.1, OpenCV 3.4.1, Numpy 1.15, and SimpleITK 2.0. The training process of the deep convolutional neural network (DCNN) model is a crucial phase where the model learns to recognize patterns and features within the training data. In this section, we will provide an overview of the key steps involved in training the DCNN model:

Data Preprocessing: Before training begins, the laryngoscopic images are preprocessed to ensure uniformity and compatibility with the model. This preprocessing typically involves resizing the images to a consistent resolution 512 × 512, normalizing pixel values to a common scale (0–255).

Initialization: The DCNN model is initialized with random weights or pretrained weights from a model pretrained on a large dataset like ImageNet. Transfer learning from a pretrained model often accelerates convergence and boosts performance. Initially, the learning rate was 0.001, which decreased by a factor of 0.5 after every 100 epochs. The total number of epochs was 16,000. During training, this learning rate was changed to increase performance and training speed and the optimizer was ‘SGD’.

Loss Function Selection: A suitable loss function was chosen based on the nature of the classification task. For binary classification (LSCC vs. benign), a common choice is binary cross-entropy loss. For multi-class problems, categorical cross-entropy may be used.

Optimizer: An optimizer, such as Adam, SGD (Stochastic Gradient Descent), or RMSprop, is employed to adjust the model’s weights during training to minimize the selected loss function. The learning rate and other hyperparameters associated with the optimizer are carefully tuned to ensure effective convergence.

Mini-Batch Training: To manage memory and computational resources efficiently, training is typically performed in mini-batches. During each training iteration, a batch of laryngoscopic images and their corresponding ground truth labels are fed into the model. The optimizer computes gradients and updates the model weights based on this mini-batch. The batch size was 64.

Backpropagation: After each mini-batch forward pass, backpropagation is used to calculate gradients with respect to the loss function. These gradients are then used to update the model’s weights in the direction that minimizes the loss.

Regularization Techniques: To prevent overfitting, regularization techniques such as dropout and L2 regularization may be applied. These methods help the model generalize better to unseen data.

Validation: During training, a separate validation dataset, distinct from the training set, is used to assess the model’s performance at regular intervals (e.g., after each epoch). This allows for early stopping if the model’s performance on the validation data starts deteriorating, preventing overfitting.

Monitoring and Logging: Key metrics such as accuracy, loss, and possibly others like precision, recall, and F1-score, are monitored and logged during training. Visualization tools and logging systems are often employed to keep track of the model’s progress.

The training process is iterative, with the model gradually learning to make accurate predictions as it updates its weights during each epoch. This process continues until the model reaches a level of performance deemed satisfactory for the given task.

In this study, we diligently followed these steps and fine-tuned hyperparameters as needed during the model training process. This study compared multiple deep learning models; all models were trained with completely consistent hyperparameters to ensure the scientificity of the comparison.

### 2.4. Statistical Analysis

In this section, we present a rigorous statistical analysis to evaluate the performance of our deep convolutional neural network (DCNN) model in the context of laryngeal cancer diagnosis based on laryngoscopic images. The assessment encompasses several key metrics, including accuracy, specificity, sensitivity, receiver operating characteristic (ROC) analysis, area under the curve (AUC), and the DeLong test.

Accuracy: Accuracy is a pivotal metric quantifying the overall classification performance of our model. It is defined as the ratio of correctly classified samples to the total number of samples. Mathematically, it can be expressed as:

Accuracy = (True Positives + True Negatives)/(True Positives + True Negatives + False Positives + False Negatives)

where TP (True Positives) denotes accurately identified laryngeal cancer cases, TN (True Negatives) represents correctly identified non-cancerous laryngeal lesions, FP (False Positives) corresponds to non-cancerous laryngeal lesions cases incorrectly identified as laryngeal cancer, and FN (False Negatives) denotes laryngeal cancer cases incorrectly classified as non-cancerous laryngeal lesions.

Specificity: Specificity assesses the model’s capability to correctly identify non-cancerous laryngeal lesions cases. It is calculated as:

Specificity = TN/(TN + FP)

Sensitivity: Sensitivity, also referred to as true positive rate or recall, measures the model’s ability to accurately detect laryngeal cancer cases. It can be calculated as:

Sensitivity = TP/(TP + FN)

Receiver Operating Characteristic (ROC) Analysis: ROC analysis is employed to visualize the model’s performance across different threshold settings. It generates an ROC curve illustrating the trade-off between sensitivity and specificity at varying thresholds.

Area Under the Curve (AUC): The AUC quantifies the overall performance of the model by calculating the area under the ROC curve. A higher AUC signifies superior discrimination, with 1 indicating perfect discrimination and 0.5 representing random chance.

DeLong Test: The DeLong test serves as a statistical tool for comparing the ROC curves of multiple classification models. It determines whether observed differences in AUC values are statistically significant, aiding in model selection and validation.

Statistical Procedure:

Accuracy, specificity, and sensitivity were computed based on the model’s predictions against the ground truth labels within the dataset. ROC analysis was executed to construct the ROC curve, and the AUC was quantified as a holistic measure of the model’s discriminatory capacity. To discern any significant distinctions in performance among different models or model variants, the DeLong test was applied. This statistical test ascertained whether variations in AUC values were statistically meaningful. In the discussion section, the outcomes of these meticulous statistical analyses offer valuable insights into the effectiveness of our DCNN model in the diagnosis of laryngeal cancer from laryngoscopic images. Additionally, they enable the assessment of potential performance disparities between our model and alternative models or variations in the classification task.

## 3. Results

**Figure 4:**
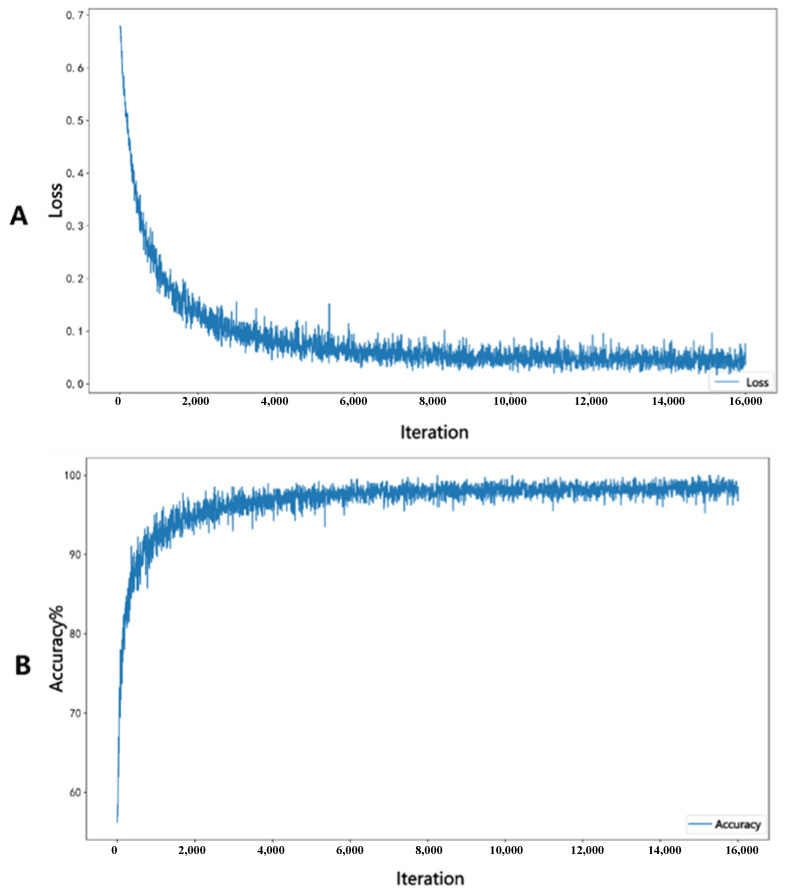
Figure (**A**) represents the loss decrease curve, while Figure (**B**) represents the accuracy change curve.

In the process of training Densenet201, as the number of iterations increases, the loss function continuously decreases and the accuracy of the internal validation group continuously improves, as shown in Figure 4. Figure 4A represents the loss decrease curve, while Figure 4B represents the accuracy change curve.

**Figure 5:**
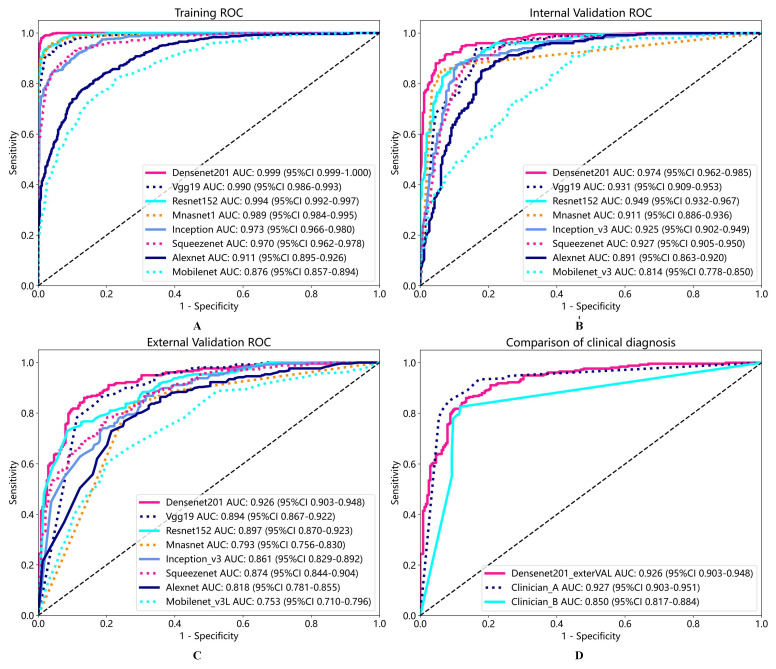
ROC of multi-model training process (**A**), internal validation (**B**), external validation (**C**), and comparison of ROC between external validation and clinical models in Densenet201 (**D**).

**Figure 6:**
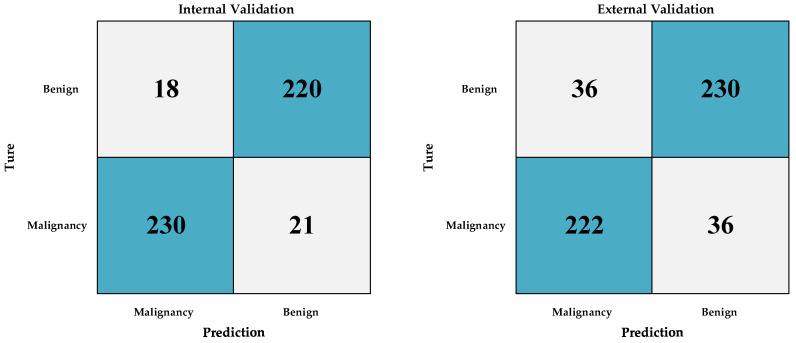
Confusion matrix between internal validation group and external validation group of Densenet201.

To verify the performance of our model, we trained multiple deep learning models simultaneously using the same batch of data and conducted performance tests. At the same time, we invited clinical doctor A with 30 years of experience in laryngoscopy diagnosis and clinical doctor B with 10 years of experience in laryngoscopy diagnosis to diagnose the external validation group’s laryngoscopy images. Based on personal experience, the scores were scored from 0 to 1. The greater the likelihood of malignancy, the closer the score was to 1. We also analyzed the accuracy and AUC of the scores given by the two doctors. The specific results of performance testing for various deep learning models and clinical diagnostic models are shown in Table 4 and Table 5 and Figure 5. Confusion matrices between the internal validation group and the external validation group of Densenet201 are shown in Figure 6. We offer a comprehensive analysis of the performance of different models, including Densenet201, Alexnet, Inception v3, Mnasnet, Mobilenet v3, Resnet152, Squeezenet1, Vgg19, clinician A, and clinician B, in the context of diagnosing laryngeal cancer based on laryngoscopic images. The evaluation metrics encompass accuracy, AUC, 95% confidence intervals (CI), sensitivity, specificity, and recall, which were computed for each model across three cohorts: Train, Internal Validation, and External Validation. We also provide comparisons with the performance of clinician assessments (clinician A and clinician B) on the External Validation cohort [41,42].

Densenet201 demonstrated excellent performance across all cohorts, achieving an accuracy of 98.5% in the Train cohort, 92.0% in the Internal Validation cohort, and 86.3% in the External Validation cohort. The AUC values for Densenet201 consistently ranked high, with 99.9% in the Train cohort, 97.4% in the Internal Validation cohort, and 92.6% in the External Validation cohort, indicating its strong discriminatory ability. Importantly, the model exhibited a sensitivity of 98.9% and specificity of 98.2% in the Train cohort, ensuring accurate detection of both positive and negative cases. These results highlight Densenet201 as the leading model in this study, showcasing its potential as a valuable diagnostic tool for laryngeal cancer.

In contrast, other models, including Alexnet, Inception v3, Mnasnet, Mobilenet v3, Resnet152, Squeezenet1, and Vgg19, while showing respectable performance in the Train cohort, demonstrated varying degrees of performance degradation in the External Validation cohort. These models generally exhibited lower sensitivity and specificity compared to Densenet201, indicating a reduced ability to accurately identify laryngeal cancer cases.

Additionally, the results indicate that clinician A and clinician B, while achieving competitive sensitivity and specificity values, clinician B displayed a lower accuracy compared to Densenet201, particularly in the External Validation cohort. Clinician A and Densenet201 exhibit very similar performance indicators. This suggests that the deep learning model, Densenet201, can serve as a valuable complementary tool for clinicians in the accurate diagnosis of laryngeal cancer [43].

It is worth noting that the DeLong test was conducted to assess the statistical significance of performance differences between Densenet201 and clinician models. As shown in Table 6, the *p*-values obtained from these comparisons serve as critical statistical indicators of the dissimilarity or similarity in performance between the evaluated groups. These statistical comparisons provide insights into the relative performance of Densenet201 and the clinicians (clinician A and clinician B) in the diagnosis of laryngeal cancer. While Densenet201 shows comparable performance to clinician A, it demonstrates a statistically significant difference in performance compared to clinician B. Moreover, clinician A and clinician B themselves exhibit significant differences in their diagnostic assessments. These findings underscore the importance of considering Densenet201 as a complementary tool to clinical expertise, particularly in cases where different clinicians may have varying levels of diagnostic accuracy [44].

## 4. Discussion

Our study aimed to assess the efficacy of deep learning models in diagnosing laryngeal cancer using laryngoscopic images, comparing them with experienced clinicians. Notably, our Densenet201 model exhibited exceptional sensitivity and specificity, rivaling a highly experienced clinician. It surpassed clinicians with a decade of experience, offering consistent interpretation, high sensitivity, specificity, and rapid processing speed, making it invaluable for dynamic detection in regions with limited otolaryngologists [45].

We conducted a comprehensive assessment, evaluating various deep learning models alongside two experienced clinicians (clinician A and clinician B) in laryngeal cancer diagnosis using laryngoscopic images. Densenet201 emerged as the leading model, boasting 98.5% accuracy in the Train cohort, 92.0% in Internal Validation, and 86.3% in External Validation. It consistently delivered high AUC values, demonstrating remarkable sensitivity and specificity, highlighting its potential as a diagnostic tool [24].

Conversely, other deep learning models like AlexNet, Inception v3, MnasNet, MobileNet v3, ResNet152, SqueezeNet1, and VGG19, while performing respectably in the Train cohort, exhibited varying degrees of performance degradation in External Validation. They generally showed lower sensitivity and specificity compared to Densenet201, emphasizing the latter’s superior accuracy in identifying laryngeal cancer cases [37].

Densenet201’s unique architecture, characterized by dense connectivity, makes it a powerful tool for image recognition tasks. It efficiently reuses features, addresses gradient vanishing, and maintains competitive performance with fewer parameters compared to other architectures. It consistently outperformed other models and even rivaled experienced clinicians, particularly in External Validation. This underscores deep learning models’ potential to provide more accurate and consistent diagnoses, especially where clinician accuracy varies. The statistical analysis, including the DeLong test, confirmed the significance of performance disparities, highlighting the importance of integrating deep learning in laryngeal cancer diagnosis [38].

While these findings are promising, further research opportunities exist. Expanding the dataset with more cases, including precancerous lesions, is a promising avenue. Additionally, optimizing the deep learning model with improved algorithms can enhance its performance.

## 5. Conclusions

In summary, our study demonstrates that deep learning models, particularly Densenet201, offer exceptional accuracy and can complement clinicians in laryngeal cancer diagnosis. The significance of performance disparities underscores the potential of integrating deep learning into laryngeal cancer diagnosis. The limitations of this study include the relatively small dataset of cases and insufficient segmentation. A logical next phase of this research will involve the creation of a substantial database of laryngoscopic images through a collaborative effort among multiple centers. However, future research should focus on dataset expansion and algorithm optimization. Furthermore, this database will encompass a more extensive range of groups, including those with precancerous lesions, which currently remain underreported in the literature with regard to the AI diagnosis of early-stage laryngeal cancer. In future work, we will continuously expand our sample library and conduct research on early laryngeal cancer data, shifting our focus to specifically identifying precancerous lesions in laryngeal cancer. This research direction will pose new challenges to the sample size and algorithm difficulty of our research.

Our study demonstrates that Densenet201 is a highly accurate and reliable tool for the computer-aided diagnosis of laryngeal cancer based on laryngoscopic images. Furthermore, our findings highlight the complementary nature of deep learning and clinical expertise, providing a foundation for improved diagnostic accuracy and patient care in the field of laryngeal cancer diagnosis.

## Figures and Tables

**Table 1:** Details of the image sets and clinical characteristics.

	Benign					Malignancy				
	Cases	Male	Female	Age	Images	Cases	Male	Female	Age	Images
Training cohort	127	53	74	45 ± 12.3	677	105	102	3	52 ± 8.6	564
Internal validation cohort	53	20	33	46 ± 12.8	238	45	44	1	52 ± 9.6	251
External validation cohort	53	24	29	41 ± 11.2	266	45	44	1	53 ± 9.1	258

**Table 2:** The detailed pathological results table of benign cases.

Histopathological Results	Epiglottic Cyst	Granulomatous	Laryngeal Keratosis	Papiloma	Tuberculosis	Vocal Fold Cyst	Vocal Polyp	Total
No. of cases	7	4	5	4	6	1	206	233

**Table 3:** Detailed parameters of Densenet201.

Layers	Parameters	Output Size
Convolution	7 × 7 *conv*, stride 2	112 × 112
Dense Block 1	1×1 conv3×3 conv × 6	56 × 56
Transition Layers 1	1 × 1 *conv*	56 × 56
	2 × 2 average pool, stride 2	28 × 28
Dense Block 2	1×1 conv3×3 conv × 12	28 × 28
Transition Layers 2	1 × 1 *conv*	28 × 28
	2 × 2 average pool, stride 2	14 × 14
Dense Block 3	1×1 conv3×3 conv × 48	14 × 14
Transition Layers 3	1 × 1 *conv*	14 × 14
	2 × 2 average pool, stride 2	7 × 7
Dense Block 4	1×1 conv3×3 conv × 32	7 × 7
Classification Layers	7 × 7 global average pool	1 × 1
	Fully-connected, softmax	

**Table 4:** Performance analysis results of multiple models.

Model Name	Acc	AUC	95% CI	Sensitivity	Specificity	Cohort
Densenet201	0.985	0.999	0.998–0.999	0.989	0.982	Train
	0.920	0.974	0.962–0.985	0.916	0.924	Internal Validation
	0.863	0.926	0.903–0.948	0.860	0.865	External Validation
Alexnet	0.826	0.911	0.895–0.926	0.810	0.839	Train
	0.835	0.891	0.863–0.919	0.853	0.817	Internal Validation
	0.758	0.818	0.781–0.855	0.767	0.757	External Validation
Inception v3	0.908	0.973	0.965–0.980	0.847	0.958	Train
	0.883	0.925	0.902–0.948	0.876	0.897	Internal Validation
	0.780	0.861	0.829–0.892	0.868	0.712	External Validation
Mnasnet	0.959	0.989	0.983–0.994	0.958	0.961	Train
	0.895	0.911	0.885–0.936	0.853	0.969	Internal Validation
	0.780	0.793	0.755–0.829	0.822	0.989	External Validation
Mobilenet v3	0.793	0.876	0.856–0.894	0.821	0.770	Train
	0.728	0.814	0.778–0.850	0.908	0.555	Internal Validation
	0.698	0.753	0.710–0.796	0.605	0.798	External Validation
Resnet152	0.960	0.994	0.992–0.996	0.948	0.970	Train
	0.887	0.949	0.932–0.966	0.861	0.913	Internal Validation
	0.819	0.897	0.870–0.923	0.729	0.932	External Validation
Squeezenet1	0.910	0.970	0.961–0.977	0.937	0.888	Train
	0.874	0.927	0.904–0.950	0.884	0.870	Internal Validation
	0.790	0.874	0.844–0.903	0.783	0.798	External Validation
Vgg19	0.944	0.990	0.985–0.993	0.942	0.946	Train
	0.885	0.931	0.909–0.952	0.936	0.870	Internal Validation
	0.841	0.894	0.866–0.922	0.868	0.915	External Validation

**Table 5:** Performance comparison of clinician models and Densenet201.

Model Name	Acc	AUC	95% CI	Sensitivity	Specificity	Data Cohort
Densenet201	0.863	0.926	0.9030–0.9482	0.860	0.866	External Validation
Clinician A	0.881	0.927	0.9029–0.9506	0.849	0.969	External Validation
Clinician B	0.853	0.85	0.8175–0.8835	0.826	0.972	External Validation

**Table 6:** Delong test results of clinician models and Densenet201.

Group	*p*-Value
Densenet201 and Clinician A	0.0891 > 0.05
Densenet201 and Clinician B	0.0205 < 0.05
Clinician A and Clinician B	0.0191 < 0.05

## Data Availability

The original data can be provided with the approval of the ethics committee of our unit and the consent of the corresponding author.
